# Reduced subthalamic and subthalamic-cortical coherences associated with the therapeutic carryover effect of coordinated reset deep brain stimulation

**DOI:** 10.1038/s41531-024-00797-w

**Published:** 2024-09-28

**Authors:** Lvpiao Zheng, Ziling Luo, Biswaranjan Mohanty, Sana Amoozegar, Luke A. Johnson, Jerrold L. Vitek, Jing Wang

**Affiliations:** Department of Neurology, University of Minnesota, Minneapolis, MN USA

**Keywords:** Parkinson's disease, Preclinical research, Translational research

## Abstract

Coordinated reset deep brain stimulation (CR DBS), a promising treatment for Parkinson’s disease (PD), is hypothesized to desynchronize neuronal populations. However, little in vivo data probes this hypothesis. In a parkinsonian nonhuman primate, we found that subthalamic CR DBS suppressed subthalamic and cortical-subthalamic coherences in the beta band, correlating with motor improvements. Our results support the desynchronizing mechanism of CR DBS and propose potential biomarkers for closed-loop CR DBS.

## Introduction

Movement dysfunction in Parkinson’s disease (PD) has been associated with exaggerated beta oscillatory power^1–3^ and increased coherence^4–6^ in the basal-ganglia-thalamocortical (BGTC) network. Therapeutic high frequency deep brain stimulation (DBS) has been shown to effectively suppress these abnormal synchronies^5–7^. Nonetheless, DBS can be accompanied with adverse effects caused by current spread into unintended brain regions when the lead is sub-optimally placed^8,9^, as well as high battery consumption requiring either frequent battery recharge or replacement.

A novel DBS strategy, i.e., coordinated reset DBS (CR DBS), has the potential to address these limitations associated with traditional DBS. CR DBS utilizes burst stimulation across different contacts of the DBS lead at a stimulation amplitude significantly lower than traditional DBS. It can produce therapeutic benefits that sustain beyond the stimulation duration, i.e., carryover effect^10–12^. This DBS pattern was designed and hypothesized to counteract excessive synchronization in neuronal populations by resetting the phases of the oscillatory activities from neuronal sub-populations^13,14^. An in vitro study supported this hypothesis by demonstrating reduced phase synchrony of hippocampal local field potentials (LFPs), resulting in reduced epileptiform neural activity during and after CR DBS^15^. Another pilot clinical study demonstrated a reduction in subthalamic beta and theta oscillatory power after therapeutic CR DBS in the subthalamic nucleus (STN)^11^. In addition, a study in the nonhuman primate (NHP) model of PD reported reduced cortical and subcortical beta oscillatory power as well as cross-hemisphere cortical coherence immediately and days after CR DBS^16^. One common limitation of these studies is that the direct relationship between changes in neural activity (e.g., oscillatory power and coherence) in the BGTC network and improvements in motor function associated with CR DBS was not investigated.

In this study, we investigated changes in the oscillatory power within and coherence between subregions in the STN, as well as STN, primary motor (M1) and premotor (PM) cortical areas after STN CR DBS in a NHP model of PD. Moreover, we studied the relationship between these changes and carryover motor improvements associated with CR DBS.

**Fig. 1:**
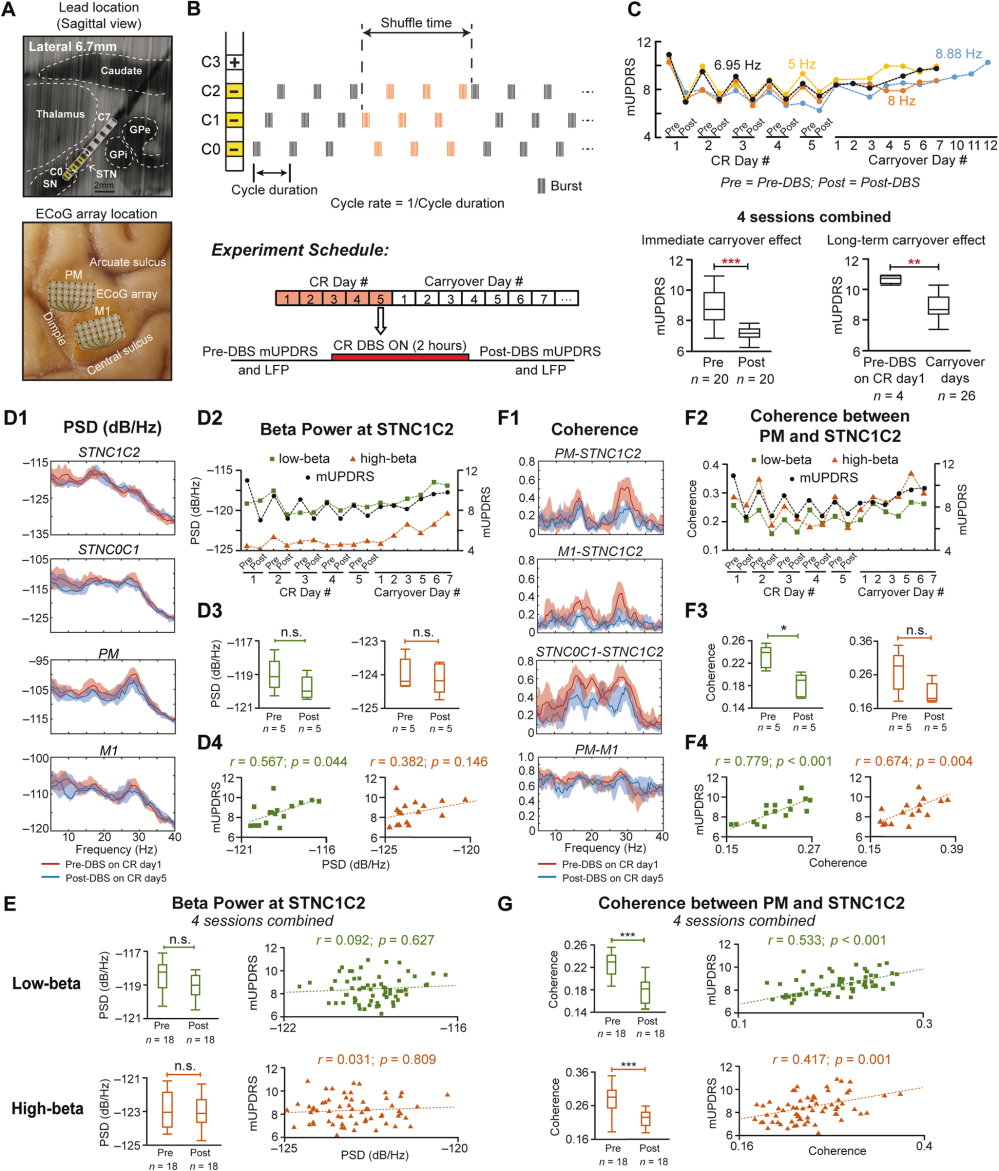
Carryover effects of CR DBS on the power and coherence in the low and high beta bands, and their correlations with the mUPDRS scores. **A** Histological locations of the DBS lead (top) and two ECoG arrays (bottom). **B** CR DBS pattern (top) and the experiment schedule (bottom). **C** Top: Daily mUPDRS scores during four CR sessions. Bottom: Comparison of mUPDRS scores revealing immediate and long-term carryover motor benefits of STN CR DBS (Wilcoxon test, **p < 0.01, ***p < 0.001), *n* = sample number. **D1**−**D4**, **F1**−**F4** demonstrate neural data from the CR session using a cycle rate of 6.95 Hz. Colors of green and orange indicate low and high beta respectively. Differences between LFPs from DBS contacts C1 and C2, and C0 and C1, were termed as STNC1C2 and STNC0C1, respectively. **D1** Power spectral densities (PSDs) of the LFPs from individual brain areas pre- and post- 5 days of CR DBS. **D2** Daily changes in STN low and high beta power as well as the mUPDRS scores. **D3** Immediate changes in low and high beta power after CR DBS (Wilcoxon test, *n.s*. = not significant). **D4** Correlation between the mUPDRS scores and low/high beta power for the entire session. **E** Data from all four CR sessions illustrating the immediate changes in low and high beta power after CR DBS (left, Wilcoxon test, *n.s*.) and the correlations between the mUPDRS scores and low/high beta power (right). **F1** Coherences between LFPs from STN, PM and M1 pre- and post- 5 days of CR DBS. **F2** Daily changes in low and high beta coherences between PM and STN as well as the mUPDRS scores. **F3** Immediate changes in low and high beta coherences after CR DBS (Wilcoxon test, *p < 0.05). **F4** Correlation between the mUPDRS scores and low/high beta coherences for the entire session. **G** Data from all four CR sessions illustrating the immediate changes in low and high beta coherences after CR DBS (left, Wilcoxon test, ***p < 0.001) and the correlation between the mUPDRS scores and low/high beta coherences (right).

Prior to all CR evaluations, the animal was in a stable moderately parkinsonian state as indicated by a clinical rating scale (mUPDRS: 10.6 ± 0.3). STN CR DBS induced both immediate and long-term carryover improvements in the mUPDRS scores across all four sessions using different CR cycle rates (Fig. 1C, top). The reduction in the mUPDRS score accumulated across 5 CR DBS days. Following discontinuation of CR DBS the score gradually returned to the baseline level 4–12 days after stimulation cessation. Statistical analysis, including data from all four CR sessions, revealed significant motor improvement immediately following CR DBS (Wilcoxon test, *z* = −4.91, ***p < 0.001) and days after CR DBS was discontinued (Wilcoxon test, *z* = −3.11, **p < 0.01) (Fig. 1C, bottom).

Given that 7 Hz cycle rate has been used in CR DBS pattern in multiple previous studies^10,12,16^, the CR session using the 6.95 Hz cycle rate is taken as an example to demonstrate the effect of CR DBS on neural activities. During this CR session, although oscillatory activities in both low (10–20 Hz) and high (21–35 Hz) beta bands were observed, there was very little change in beta power in STN, M1 and PM immediately after 5 CR days compared with the pre-DBS baseline level (Fig. 1D1). Taking beta oscillatory power within the STN (STNC1C2, the difference between LFPs from DBS contacts C1 and C2) as an example, changes in low beta power across days showed a similar trend as the mUPDRS scores, while those in high beta power demonstrated a different trend (Fig. 1D2). When comparing pre- and post-DBS power from 5 CR days, no significant change was observed in low or high beta power immediately after CR DBS (Fig. 1D3; Wilcoxon test, *n.s*.), despite a trend of decrease in low beta power. Low beta power was correlated with the mUPDRS scores while high beta power did not demonstrate any correlation (Fig. 1D4).

With data from all four CR sessions combined, no significant difference was observed between pre- and post-DBS STNC1C2 power in either low or high beta band (Fig. 1E, left; Wilcoxon test, *n.s*.). Additionally, neither low nor high beta power at STNC1C2 exhibited linear correlation with mUPDRS scores (Fig. 1E, right). Similar results were observed for beta power from STNC0C1 (the difference between LFPs from DBS contacts C0 and C1), PM, and M1 (Table 1, top). Specifically, low and high beta power were not suppressed immediately after CR DBS on CR days. None of the low and high beta power was linearly correlated with the mUPDRS scores, except for the low beta power from PM with a weak correlation.

**Table 1:** Statistical analysis for power and coherence changes immediately following CR DBS, and correlations between the power/coherences and the mUPDRS scores (*p* values corrected using the FDR method)

Power	STNC1C2		STNC0C1		PM		M1	
	low beta	high beta	low beta	high beta	low beta	high beta	low beta	high beta
**Immediate change (*z*)**	−2.36	−0.016	0.079	0.965	−2.61	−0.395	−1.06	0.744
**Immediate change (*p*)**	0.074	0.987	0.987	0.669	0.072	0.923	0.669	0.732
**Correlation (*r*)**	0.092	0.031	−0.147	−0.197	0.363	0.205	0.195	−0.076
**Correlation (*p*)**	0.627	0.809	0.394	0.247	0.026*	0.247	0.247	0.628

Coherence	PM-STNC1C2		M1-STNC1C2		STNC0C1-STNC1C2		PM-M1	
	low beta	high beta	low beta	high beta	low beta	high beta	low beta	high beta
**Immediate change (*z*)**	−4.54	−3.91	−4.00	−2.04	−4.16	−4.82	0.142	−0.680
**Immediate change (*p*)**	**<** 0.001***	**<** 0.001***	**<** 0.001***	0.055	**<** 0.001***	**<** 0.001***	0.887	0.567
**Correlation (*r*)**	0.533	0.417	0.499	0.277	0.431	0.378	0.108	0.229
**Correlation (*p*)**	**<** 0.001***	0.001***	**<** 0.001***	0.035*	0.001**	0.003**	0.394	0.079

Bolded *p* values indicate significant changes or correlations.

*p < 0.05, **p < 0.01,***p < 0.001.

In contrast, during CR session with 6.95 Hz cycle rate, reductions in the coherences in the low and high beta bands were observed after 5 days of CR DBS between STN and cortical areas as well as between STN subareas, but not between cortical areas (Fig. 1F1). Taking PM-STNC1C2 coherence as an illustration, PM-STNC1C2 coherence in both low and high beta bands across days showed similar trends of changes with the mUPDRS scores (Fig. 1F2). Specifically, low beta PM-STNC1C2 coherence significantly decreased immediately after CR DBS (Fig. 1F3, left; Wilcoxon test, *z* = −2.51, *p* < 0.05), while no significant change was observed in high beta coherence although a trend of reduction was clear (Fig. 1F3, right; Wilcoxon test, *n.s*.). Additionally, PM-STNC1C2 coherences in both low and high beta bands were positively correlated with the mUPDRS scores (Fig. 1F4).

PM-STNC1C2 coherences across four CR sessions were suppressed in both low and high beta bands immediately after CR DBS (Fig.1G, left; Wilcoxon test, *z* = −4.54 and *z* = −3.91, *p* < 0.001). Both low and high beta coherences were positively correlated with the mUPDRS scores (Fig. 1G, right). In addition, significant reductions immediately following CR DBS were also observed for M1-STNC1C2 low beta, and STNC0C1-STNC1C2 low and high beta coherences (Table 1, bottom). PM-M1 coherences were not suppressed by CR DBS in either low or high beta bands. Moreover, M1-STNC1C2 and STNC0C1-STNC1C2 coherences in both low and high beta bands were positively correlated with the mUPDRS scores (Table 1, bottom), while neither low nor high beta PM-M1 coherences were correlated with the mUPDRS scores.

Excessive synchrony of neuronal activity in the BGTC network, e.g., beta oscillatory power and coherence, has been associated with motor impairments of PD^1–4,17,18^. Symptoms alleviated by traditional DBS were found associated with the suppression of beta power or coherence^5–7,19–21^. Meanwhile, in vivo investigation of the mechanism underlying the therapeutic effect of CR DBS has been extremely limited. A reduction of the STN oscillatory power in the theta and beta band after STN CR DBS was observed in a pilot clinical study^13^ and a preclinical study in the NHP model of PD^19^. Our results, however, demonstrate no significant change in low or high beta power in the STN immediately after STN CR DBS, nor in M1 or PM. This questions the role of changes in the oscillatory power within individual nuclei in response to therapeutic CR DBS. Nevertheless, we observed significant reductions in subcortical-subcortical and cortical-subcortical coherences after CR DBS. Previous work also demonstrated reduced cross-hemisphere cortical coherence following STN CR DBS^19^, however, whether these changes in neural activity correlated with motor improvement was not studied. Our data provide a direct correlation between the severity of parkinsonian motor signs and neural activities. The reduced subcortical-subcortical and cortical-subcortical coherences that correlated with motor improvements might play a more important role than oscillatory power in the mechanism of CR DBS. Moreover, these results suggest both local, i.e., within the STN region, and network, e.g., STN-M1 and STN-PM, desynchronization induced by STN CR DBS.

The carryover benefits observed in our study also indicate a different mechanism of CR DBS compared with traditional DBS which is not associated with long-term carryover effects. Traditional DBS, using a relatively high stimulation amplitude, is hypothesized to decouple soma activity from axonal output, inhibiting local somatic activity and activating axonal output^22–24^. These effects could replace the intrinsic activity of the stimulated brain region as well as downstream nuclei thus overriding the pathological neuronal discharges^24–26^. Though plasticity can be altered by traditional DBS^27,28^, therapeutic latencies of DBS for different PD symptoms are relatively short, with reappearance of tremor within seconds, rigidity within minutes and bradykinesia within hours from DBS cessation^29,30^. In contrast, CR DBS, delivering stimulation at a significantly lower amplitude to neuronal subpopulations, is hypothesized to phase-desynchronize local neuronal subpopulations and downregulate network synaptic connectivity^31,32^. Therefore, instead of “masking” the stimulated area (e.g., suppressing beta oscillation), CR DBS may function by reducing the coherence of neuronal subpopulations in the beta band while maintaining a level of beta oscillatory power. The long-lasting suppression of the cortical-subcortical coherence might also indicate a relatively stable, desynchronized basal-ganglia-cortical circuit induced by the 5-day CR treatment.

Some limitations should be noted while interpreting our data. Although repeated CR sessions were evaluated, different cycle rates were used and data were from one subject. Additional evaluations in more subjects will be needed to validate our findings. Multiple Parkinsonian motor signs were assessed in the study, however, they were subjectively evaluated using a clinical rating scale. Future incorporation of objective, quantitative measurements of motor signs, e.g., kinematic data obtained using markerless motion capture techniques, might facilitate correlation of neural features with specific motor impairments. Despite these limitations, our results provide crucial insights into the action mechanism underlying the carryover effect of CR DBS and propose potential biomarkers for further optimizing CR DBS and developing closed-loop CR DBS approaches.

## Methods

### Animal procedures and experiments

Animal care complied with the National Institutes of Health Guide for the Care and Use of Laboratory Animals and all procedures were performed under a protocol approved by the Institutional Animal Care and Use Committee of the University of Minnesota.

An adult female NHP (Macaca mulatta, 8.2 kg) was rendered moderately parkinsonian through intramuscular administration of the neurotoxin 1-methyl-4-phenyl-1,2,3,6-tetrahydropyridine (MPTP, 0.3–0.4 mg/kg, 8 injections). The severity of parkinsonian motor signs was assessed using a Unified Parkinson’s Disease Rating Scale modified for NHP use (mUPDRS). The mUPDRS evaluates five key parkinsonian features (rigidity, bradykinesia, akinesia, tremor, and food retrieval) on a 4-point scale (0–3; 0=unimpaired, 3=severe) with ratings collected for both the upper and lower limbs (maximum possible score = 27; moderate stage: 10–18). The animal was implanted with a DBS lead in the STN region and 2 electrocorticography (ECoG) arrays over M1 and PM. The implantation procedures have been demonstrated in our previous study^12,33^. Briefly, a cephalic chamber was placed on the skull oriented to target the STN based on CT and MRI imaging. Microelectrode recording and stimulation techniques were used to map the sensorimotor region and borders of the STN, following which an 8-contact DBS lead (NuMed Inc., 0.63 mm diameter, 0.5 mm contact height, and 0.5 mm space between contacts) was implanted. Two 32-channel ECoG arrays (NeuroNexus) were placed during an aseptic surgery based on anatomical landmarks. The DBS lead and ECoG array cables and connectors were housed in two dry chambers allowing access of stimulation and recording. At the end of the study, histology was performed with 40 μm coronal sections imaged and visualized to show the DBS lead location (Fig. 1A, top) and cortical surface imaged to show the ECoG array locations (Fig. 1A, bottom).

STN CR DBS (Fig. 1B, top) was evaluated for four times utilizing four different cycle rates (8.88, 8, 6.95, and 5 Hz), with the rest of stimulation parameters set at: stimulation intensity = 0.16 mA, pulse width = 120 μs, intra-burst rate = 150 Hz, shuffle time = 10 s. These cycle rates were selected around the “standard” cycle rate of 7 Hz reported in previous studies^10,12^ and are the actual frequencies the device (Boston Scientific Precision Spectra) was able to generate to approximate the desired frequencies (i.e., 6.95 Hz approximates 7 Hz and 8.88 Hz approximates 9 Hz). In each evaluation session, CR DBS was delivered through four bottom contacts (cathodes: C0-C2, anode: C3) of the DBS lead over five consecutive days for two hours per day (Fig. 1B, bottom). mUPDRS was assessed and resting state LFPs (5 min) from STN, M1, and PM were recorded pre and post stimulation on CR DBS days, as well as on carryover days. To avoid the overlap of the effects between two adjacent CR DBS sessions, mUPDRS was assessed daily after each session and the following CR session was not initiated until the mUPDRS score returned to baseline for at least four consecutive days. LFPs were recorded using a multi-channel neuronal recording system (Tucker Davis Technology) at ~24 KHz. Bipolar LFPs measuring the differences between LFPs from adjacent DBS contacts (STNC1C2 and STNC0C1) and mean LFPs from the PM and M1 ECoG arrays were used for subsequent analysis in MATLAB.

### Data analysis

LFPs were filtered (< 762 Hz) and down sampled to ~3 KHz for analysis following which the resting and alert state data were identified by analyzing videos monitoring the animal’s eyes and body movements. These LFP data were segmented into 5-second epochs for power and coherence analysis. Power spectral density (PSD) and the magnitude-squared coherence of LFPs was calculated using the MATLAB function pwelch and mscohere, respectively, using a 2048 Hamming window, 1024 overlapped samples and 8192 discrete Fourier transform points leading to a 0.3725 Hz frequency resolution. The PSD and coherence analysis involved LFPs from STN, M1, and PM in both low and high beta frequency bands. The mean power/coherence in the low (10–20 Hz) and high (21–35 Hz) beta bands was calculated for each 5 s epoch, and the median of these mean power/coherence values was computed for each LFP recording. These median values pre and post DBS on CR days were used to evaluate the immediate changes in low and high beta power/coherence after CR DBS, and those from all LFP recordings across both CR and carryover days were used to investigate the correlation between the mUPDRS scores and power/coherences. The neural data from the last two CR days in the session using the 8.88 Hz cycle rate were excluded for analysis due to excessive noise in the data.

Statistical analysis was performed in MATLAB. Wilcoxon test was used to compare the mUPDRS scores between the pre and post CR DBS conditions, as well as between the baseline and carryover days. Wilcoxon test was also used to compare the beta power and coherence between the pre and post CR DBS conditions. Linear correlation analysis was used to investigate the relationship between the mUPDRS scores and power/coherences. False discovery rate (FDR) approach was used to correct *p* values from multiple Wilcoxon tests and linear correlation calculations.

## Data Availability

Data supporting the findings of this study are available from the corresponding author upon reasonable request.
